# Using GIS to establish a public library consumer health collection

**DOI:** 10.1186/1742-5581-1-3

**Published:** 2004-11-18

**Authors:** Elizabeth M LaRue

**Affiliations:** 1 McCormick Educational Technology Center, Library of Rush University, Rush University, 600 S. Paulina, Chicago, IL 60622 USA

## Abstract

**Background:**

Learning the exact demographic characteristics of a neighborhood in which a public library serves, assists the collection development librarian in building an appropriate collection. Gathering that demographic information can be a lengthy process, and then formatting the information for the neighborhood in question becomes arduous.

As society ages and the methods for health care evolve, people may take charge of their own health. With this prospectus, public libraries should consider creating a consumer health collection to assist the public in their health care needs. Using neighborhood demographic information can inform the collection development librarians as to the dominant age groups, sex, and races within the neighborhood. With this information, appropriate consumer health materials may be assembled in the public library.

**Methods:**

In order to visualize the demographics of a neighborhood, the computer program ArcView GIS (geographic information systems) was used to create maps for specified areas. The neighborhood data was taken from the U.S. Census Department's annual census and library addresses were accumulated through a free database. After downloading the census block information from  the data was manipulated with ArcView GIS and queried to produce maps displaying the requested neighborhood demographics to view in respect to libraries.

**Results:**

ArcView GIS produced maps displaying public libraries and requested demographics. After viewing the maps the collection development librarian can see exactly what populations are served by the library and adjust the library's collection accordingly.

**Conclusions:**

ArcView GIS can be used to produce maps displaying the communities that libraries serve, spot boundaries, be it "man-made or natural," that exist prohibiting customer service, and assist collection development librarians in justifying their purchases for a dedicated consumer health collection or resources in general.

## Background

Libraries have the objective to build collections that support the communities they serve. To build a viable collection the collection development librarian, outreach/marketing librarian, and others, must determine exactly what populations reside in the neighborhood, in respect to race, spoken language, educational level, and age groups. One method collection development librarians use to gather demographic information is to physically visit the communities and integrate into the neighborhoods. They may attend events within the community in order to analyze the attendees or walk around the neighborhood to get a feel for the community. Another method for collection development is to perform an informal survey with people that visit the library and learn about their preferences and/or what they like to read or look for on the Internet. Using census information can be a third way to gather community/neighborhood information. The US Census Bureau takes a census of the United States every 10 years and publishes the results on the Internet. Taking the Census information and transforming it into a graphical format provides an objective view of the communities surrounding a library. One way to visualize the census data is through the use of GIS, Geographic Information Systems. GIS are databases arranged by spatial coordinates that, when programmed, can produce maps [1]. The US Geological Survey office defines GIS as "a computer system capable of capturing, storing, analyzing, and displaying geographically referenced information; that is, data identified according to location [2]."

Many disciplines have used GIS and many large academic libraries even support GIS by establishing a GIS department and employing a librarian to specialize in GIS information. In the field of library science articles have been published describing the establishment of GIS departments in libraries and what GIS librarians do [3-7], but little has been published describing how a library has used GIS.

Governments use GIS to visualize land use planning, tax appraisal, utility and infrastructure planning and more [8]. Businesses have used GIS for real estate analysis, marketing, and demographic analysis [8]. Based on this approach, a research project was developed to test the use of GIS in collection development for a large metropolitan public library system.

Building a collection for a library has become a sophisticated art. Johnson states that "collection building consists of four steps: identifying the relevant items, assessing the item to decide if it is appropriate for the collection and evaluating its quality, deciding to purchase, and preparing an order [9]." Each step in the process of collection development produces a layer of difficulties. In order to get to these steps a collection development librarian must be aware of their audiences. To name a few necessary pieces of information, the librarian must know the predominate age of their clients, their reading genre, their reading level, their culture, and their preferred language [9]. After one has gathered community information then the decision of what to purchase for use by the community, can be initiated and carried out [10].

This paper reports the steps taken to use GIS in assisting a large metropolitan public library system in visualizing neighborhoods that surround branch libraries in order to make an educated decision on whether a dedicated consumer health collection should be established to support the community. The objective of the project was to determine if GIS could be used to improve collection development.

## Methods

After comparing large library systems in major metropolitan areas, (Chicago, Los Angeles, and New York City) Chicago was selected as a convenient sample to be mapped with GIS because of its size, number of public libraries, their geographic distribution within the urban area, and ease of access to data. Compared to other large cities, the New York Public Library system has 86 libraries and Los Angeles has 67 public libraries [11]. The city of Chicago has 77 public libraries with one central library.

With the selection of the city of Chicago, the next step was to create a map of the metropolitan area. ESRI ArcGIS v9.0 geographic information software (GIS) [12] was selected as the GIS application. The software permits data manipulation and mapping capabilities. There are four components of ArcGIS that work together to give a high level of functionality to the program: ArcReader, ArcView, ArcEditor and ArcInfo. By inserting data files into ArcEditor the datum may convert into a graphical representation. Once a base map has been created with geographic boundaries, locations may be established to show distances within the map.

In order to create the city map of Chicago, data files were obtained from the U.S. Census Bureau website. The Census Bureau produces TIGER (Topologically Integrated Geographic Encoding and Referencing) Files available for free to the public [13]. These files contain geographic structures, such as streets, highways, and addresses, for tabulation and dissemination. To set the boundary lines of metropolitan Chicago, Cook County (the county in which the city of Chicago resides) was selected as the source for the data file [14]. This TIGER file contained the street map for the city of Chicago.

The next step required mapping distances between neighborhoods and neighborhood demographics. In order to achieve this, files were obtained from the U.S. Census Bureau website [15] which distributes the 2000 Census statistics in TIGER File format. Downloading the files for Census blocks in Cook County provided the necessary data to build a visual representation of neighborhoods.

The final piece of information needed to build a map showing neighborhoods in respect to public library locations, was to map the public libraries within Chicago. Because the base map of Cook County was a street map, addresses of public libraries were entered into ArvView to mark all the public libraries within the city of Chicago. The state of Illinois provides a database available on the Internet that permits searching types of libraries and locations of libraries, . The output of this search was imported into Excel, cleaned, and converted into a dBase file. The dBase file was imported into ArcView to coordinate the library addresses to the street addresses of the base map. This enabled the software to place an image for each public library in the city map of Chicago.

**Figure 1:**
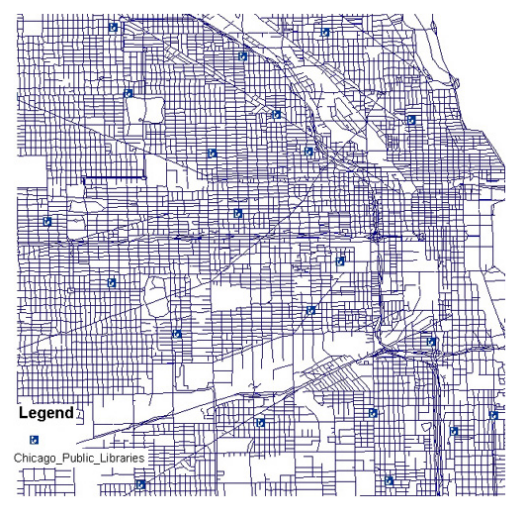
Location of public libraries in the city of Chicago

**Figure 2:**
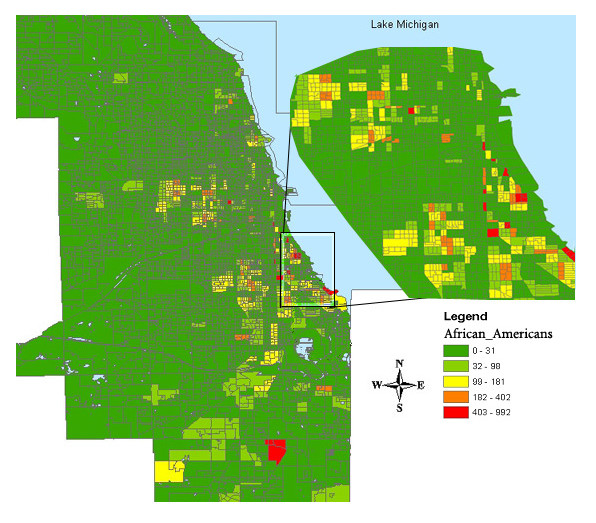
Density of African American population in southern part of Chicago

With the city of Chicago and its public libraries now in a GIS application, the location of libraries was displayed in respect to neighborhoods and distances from library to library (See Figure 1). Because the Census data was used to build the map, ArcView can be queried to show demographics of the city. This allows useful data manipulation in relation to points of interest, in this case, public libraries. Thus, ArcView was queried to show the distribution of all men and women within the city of Chicago as well as the breakdown of their age groups. The software application produced a graphic which used color schemes with a map legend. If a librarian wanted to compare the number of men in their 40s to women in their 40s, ArcView can map those fields with a well-structured query. ArcGIS was also capable of creating maps that display census ethnic groups. For example, Figure 2 shows a high percentage of African Americans in the southern neighborhoods of the city as compared to the northern part of the city. With maps such as these, a collection development librarian can graphically see the demographic breakdown of neighborhoods and build collections according to its clientele [16].

## Results

**Figure 3:**
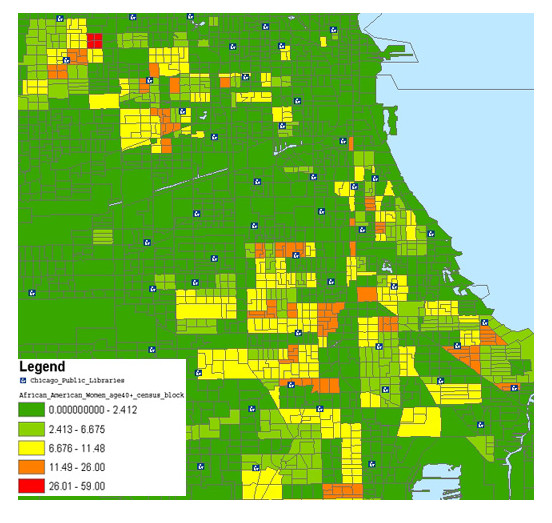
African American women in their 40s in proximity to public libraries

Fifteen maps were created showing a breakdown of each demographic with the city's public libraries. Each map provided information regarding gender, age, and race of the clientele surrounding the libraries. These maps used a combination of color schemes to graphically represent differences between groups. The maps displayed different age groups divided by ranges of age (30–39, 40–49, 50–64, 65 and up). The age ranges presented in the maps varied by query and census information. The groups analyzed were: women, men, white women (group age 40–49), African American women (group age 40–49), Asian (men/women, Latinos (men/women). The color schemes used represent the number of people that live within the census block as to the specified GIS query. In Figure 2, the numbers, 0–992 in the legend are the African Americans residing within the census block. Hence the red blocks have a large population (403–992) of African Americans as compared to the dark green areas. Figure 3 represents a query for African American women in their 40s. The results produced a map showing that 26–59 African American women in their 40s live within the shaded red blocks.

In this project ArcGIS was used to build maps of neighborhoods in the city of Chicago, display public libraries and spatially represent distances from libraries to populations. The US Census demographic information was incorporated into ArcGIS and queried to produce 15 different maps. Queries were built to display neighborhood demographics through colors showing the percentages of the population(s). Each query produced a map for a specific age or race. Once the map was produced the public libraries were added to the map and analysis was made regarding collection needs according to the neighborhood population surrounding the library.

**Figure 4:**
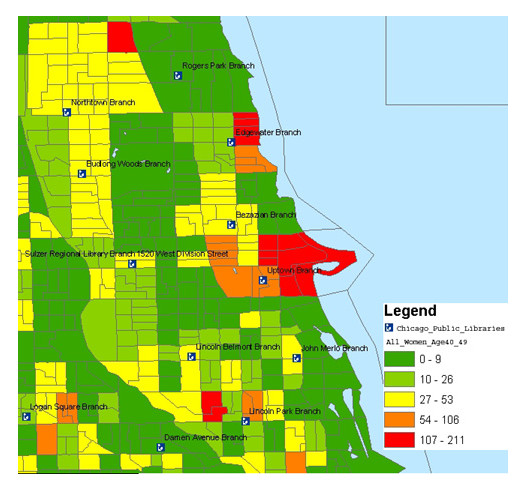
High density neighborhoods with all women in their 40s in proximity to public libraries

To decide if a public library should establish a consumer health collection, a map showing the breakdown of women in their 40s was built. As in Figure 4, the map shows a very high percentage of women 40–49 close to the Uptown Branch Library. The collection development librarian can now assess the libraries existing collection for health related materials. If there are very few resources then the collection development librarian should begin to purchase consumer health materials. If the library has a strong consumer health collection already then building maps to show the racial breakdown of the neighborhood could lead the collection development librarian to purchase culturally sensitive health materials.

## Discussion

By creating maps showing the demographic breakdown of the city, a public library can build, adjust, or verify its collection in respect to the neighborhoods it supports [17]. It is easier to define the characteristics of the library's service area clientele by making visual representations (maps) of neighborhoods surrounding a library. The neighborhood information may then be used to justify additional resource purchases, a change in collection policy and even a change in library services to fit the culture of the neighborhood. By using GIS, libraries will be capable of finding specific populations within the library's neighborhood to support or serve thus taking some of the guesswork out of collection development [17,18]. Collections may be tailored specifically to the neighborhood by using the subjective data provided with GIS.

Many studies have been conducted analyzing what people search for on the Internet and who is searching. Studies have found that women in their 40s search for and utilize health information from the Internet more than men [19-24]. Using this information to assist in collection development, a librarian would need to know how large the population of women in their 40s is in the neighborhood the library serves. Using the map created by ArcGIS showing the distribution of women in their 40s in respect to library locations can assist the collection development librarian in purchasing appropriate materials. Using the maps showing the race of women in their 40s would also assist the librarian in purchasing appropriate materials. For instance, if most women are African American then the collection development librarian would purchase materials supporting race-related health needs such as Sickle Cell Anemia, Coronary Heart Disease, Diabetes and others (See Figure 2 and 3).

### Recommendations

Using ArcGIS can assist in defining the exact make-up of the population that a library serves. By turning the census information into a graphic the library is provided a chance to see how far (physical distance) their services may reach. Library systems that have branch libraries may have one collection development librarian who buys for each library. By using ArcGIS the central library can analyze the communities surrounding each library to purchase materials appropriately without having to spend time in each library learning the environment. Purchase decisions may now be justified with hard data from ArcGIS. For example, if ArcGIS shows that the largest population surrounding a library speaks a minority language, materials should be purchased in the dominant language to best meet the needs of the community.

### Limitations

There is a fairly high learning curve to use ESRI Arcview GIS. There are five programs in ESRI Arcview and each program has separate features that must be used within the specific program to be imported into the primary map. Learning what each program does and then how to incorporate the data into the map requires hours of reading. Acquiring the necessary data to build a map requires background study of the geographic area in order to assure that the map is configured correctly. Upon attaining all necessary data for the map, one must build queries to manipulate the data according to the information need, and then configure the map accordingly.

While GIS presents a new technique to use in performing collection development, there was not a way to test the differences from a collection built using GIS information and the current methods of collection development.

In addition, research has discovered that knowing the service area radius of the library doesn't mean that people within the service area will actually use or belong to the neighborhood library [17,25]. When using GIS one must take into account geographic barriers to library access such as major highways, and railways. People are often reluctant to cross these barriers and may elect to visit another library further away with no geographic obstructions [17,25,26]. These barriers must be represented in the GIS map to fully display the areas libraries serve. The TIGER files contain some geographic structures but most will have to be hand coded. Thus, all neighborhoods should be examined in order to assure the marking of geographic barriers.

While GIS can take the census information and demonstrate the population surrounding a library, the census does not describe the specific users of the library [25]. To build a map showing a library's service area, patron addresses (information now protected by the Patriot Act) would need to be coded into GIS. Using patron zip codes of residence encompasses too much or too little area and there is no correlation between US Postal Service ZIP Codes and US Census Bureau Geography [27]. Thus, mapping or defining areas through zip codes is extremely difficult. Without patron address information, there can be no clear realization of exactly how far people will travel to a library, and if people cross-neighborhoods to use a certain library [18,25].

## Conclusions

Budget constraints will add a need for justification of purchases for a library's collection. Using GIS may provide one method to justify additions to collections. As health care changes and individuals take more control of their health, libraries need to have materials that provide valid information in an age and language appropriate format and be culturally sensitive. Branch libraries respond to the demands of the neighborhood [25] and knowing what those demands are will make an effective library [17]. GIS can assist collection development librarians located in a central library buy for branch libraries, by saving them time, and answering demographic questions about the population a library serves. Knowing details about its communities will make a more functional library within a neighborhood. To verify the use of GIS for collection development, an analysis of a library's collection should be conducted and compared with information provided from a GIS application to see if the collection satisfies the library's neighborhood.

## List of Abbreviations

GIS = geographical information systems
